# Estimating the health and economic effects of the voluntary sodium reduction targets in Brazil: microsimulation analysis

**DOI:** 10.1186/s12916-021-02099-x

**Published:** 2021-09-29

**Authors:** Eduardo Augusto Fernandes Nilson, Jonathan Pearson-Stuttard, Brendan Collins, Maria Guzman-Castillo, Simon Capewell, Martin O’Flaherty, Patrícia Constante Jaime, Chris Kypridemos

**Affiliations:** 1 Center for Epidemiological Research in Nutrition and Public Health, University of São Paulo, São Paulo, Brazil; 2 School of Public Health, Imperial College London, London, UK; 3 Department of Public Health and Policy, University of Liverpool, Liverpool, UK

**Keywords:** Sodium, Sodium reduction, Sodium targets, Health economics, Cardiovascular disease, Hypertension, Food policy, Public health, Global health

## Abstract

**Background:**

Excessive sodium consumption is one of the leading dietary risk factors for non-communicable diseases, including cardiovascular disease (CVD), mediated by high blood pressure. Brazil has implemented voluntary sodium reduction targets with food industries since 2011. This study aimed to analyse the potential health and economic impact of these sodium reduction targets in Brazil from 2013 to 2032.

**Methods:**

We developed a microsimulation of a close-to-reality synthetic population (IMPACT_*NCD-BR*_) to evaluate the potential health benefits of setting voluntary upper limits for sodium content as part of the Brazilian government strategy. The model estimates CVD deaths and cases prevented or postponed, and disease treatment costs. Model inputs were informed by the 2013 National Health Survey, the 2008–2009 Household Budget Survey, and high-quality meta-analyses, assuming that all individuals were exposed to the policy proportionally to their sodium intake from processed food. Costs included costs of the National Health System on CVD treatment and informal care costs. The primary outcome measures of the model are cardiovascular disease cases and deaths prevented or postponed over 20 years (2013–2032), stratified by age and sex.

**Results:**

The study found that the application of the Brazilian voluntary sodium targets for packaged foods between 2013 and 2032 could prevent or postpone approximately 110,000 CVD cases (95% uncertainty intervals (UI): 28,000 to 260,000) among men and 70,000 cases among women (95% UI: 16,000 to 170,000), and also prevent or postpone approximately 2600 CVD deaths (95% UI: − 1000 to 11,000), 55% in men. The policy could also produce a net cost saving of approximately US$ 220 million (95% UI: US$ 54 to 520 million) in medical costs to the Brazilian National Health System for the treatment of CHD and stroke and save approximately US$ 71 million (95% UI: US$ 17 to170 million) in informal costs.

**Conclusion:**

Brazilian voluntary sodium targets could generate substantial health and economic impacts. The reduction in sodium intake that was likely achieved from the voluntary targets indicates that sodium reduction in Brazil must go further and faster to achieve the national and World Health Organization goals for sodium intake.

**Supplementary Information:**

The online version contains supplementary material available at 10.1186/s12916-021-02099-x.

## Background

Non-communicable diseases (NCDs) are a global health problem, and unhealthy diets are among its leading drivers. Among dietary risk factors, high sodium intake was the leading cause of morbidity and mortality worldwide, accounting for some 3 million deaths and 70 million disability-adjusted life-years (DALYs) [1]. In Brazil, NCDs are responsible for 75% of all deaths, and cardiovascular disease (CVD) is the most frequent cause of death among NCDs [2]. CVD represents the most significant disease burden attributable to high sodium intake, and much of this risk is mediated through blood pressure increase [3].

The average daily sodium consumption in Brazil is approximately double the World Health Organization (WHO) [4] recommended maximum limit of 2g [5]. Thus, over 27,000 deaths from coronary heart disease (CHD) and stroke could be prevented or postponed every year if Brazilians consumed, on average, less than 2 g/day sodium [6]. However, unlike many high-income countries with “Western” type diets, only about 35% of Brazilians’ dietary sodium comes from industrialised foods and salt-based condiments, whereas over 55% comes from added table salt [7–9]. Sodium reduction policies in Brazil have therefore incorporated a set of strategies. These include health education campaigns targeted to the table salt added to foods and meals [10], and since 2011, food reformulation strategies aimed at reducing the sodium content of processed and ultra-processed products, including condiments, through voluntary upper limits for sodium content in priority food categories [11]. From 2011 to 2017, all food categories have reduced their upper limits of sodium and most have reduced their average sodium content, from 8 to 34% [12].

The objective of this study was to quantify the potential health and economic impacts of voluntary sodium reformulation in Brazil.

## Methods

We have developed IMPACT _NCD-BR_, a new microsimulation for Brazil using available local data, building on our previous experience in sodium modelling [13–16]. We used IMPACT_*NCD-BR*_ to assess the potential health and economic effects of the voluntary targets for sodium content in processed foods in Brazil over 20 years (2013–2032).

We simulated the long-term impact of reducing the sodium content of processed and ultra-processed foods through the national voluntary targets from 2013 to 2017, compared to a “no intervention” baseline scenario. We assumed that sodium reduction in foods was achieved from 2013 through 2017, that only industries officially committed to the voluntary targets would reduce the sodium content of their products (corresponding to 70% of the Brazilian market share), and that the sodium content and target compliance by industries from 2017 onwards [12] would not change. We further assumed that, given the extent of the reformulation, all individuals were exposed to the policy proportionally to their sodium intake from processed food.

### The IMPACT_NCD-BR_ model

IMPACT_NCD-BR_ is a discrete-time stochastic, dynamic, microsimulation model based on the simulated adult life course of a close-to-reality open cohort of synthetic individuals under different policy scenarios, considering the population heterogeneity and the lag times between exposures and outcomes. The data sources to the model are presented in Table 1, and the model inputs, structure, and key assumptions are detailed in Supplementary Materials Appendix.

**Table 1:** IMPACT_NCD-BR_ data sources

Parameter	Outcome	Details	Comments	Source
Population size estimates	Population	Brazilian Institute of Geography and Statistics (IBGE)	Stratified by age and sex	Brazilian Institute of Geography and Statistics (IBGE) [17]
Population projections	Population	2010–2060 Brazil population projections produced by IBGE	Stratified by year, age and sex	Brazilian Institute of Geography and Statistics (IBGE) [18]
Mortality	Deaths from CHD, stroke, and any other non-modelled causes	Underlying cause of death 2000–2016	Stratified by year, age and sex	National Mortality Information System (SIM/SUS) [19]
Sodium exposure	Exposure of individuals	National Household Budgetary Survey	Anonymised, individual-level dataset	IBGE - National Household Budgetary Survey (POF) 2008–2009 [20, 21]
Systolic blood pressure exposure	Exposure of individuals	National Health Survey	Anonymised, individual-level dataset	IBGE - National Health Survey (PNS) 2013 [22]
Effect of sodium consumption on systolic blood pressure	Systolic blood pressure	Meta-analysis/meta-regression of 103 trials	Only trials with duration >7 days were analysed	Mozaffarian et al. [3]
Reference level of sodium consumption	Ideal sodium consumption below which no excess risk was considered to occur	Evidence from ecological studies, randomised trials, and meta-analyses of prospective cohort studies	Intake levels associated with the lowest risk ranged from 614 to 2391 mg/day; in large, well-controlled randomised feeding trials, the lowest tested intake for which blood pressure reductions were clearly documented was 1500 mg/day	Mozaffarian et al. [3]
Relative risk for systolic blood pressure	CHD and stroke incidence (ICD-10: I20–I25 and I60–I69)	Pooled analysis of 2 individual-level meta-analyses	Stratified by age and sex; adjusted for regression dilution and total blood cholesterol and, where available, lipid fractions (HDL and non-HDL cholesterol), diabetes, weight, alcohol consumption, and smoking at baseline	Micha et al. [23]
	Mortality from any cause excluding CHD and stroke	Individual-level meta-analysis of 48 prospective cohort studies	Adjusted for age, sex, race or ethnicity, deprivation, smoking, diabetes, inactivity, alcohol, and obesity	Stringhini et al. [24]
Reference level of systolic blood pressure	Ideal systolic blood pressure below which no excess risk was considered to occur	Evidence from randomised trials of antihypertensive drugs and the INTERSALT study	There may be health benefits by lowering systolic blood pressure down to 110 mm Hg	Singh et al. [25]
Disease costs	Public hospitalisation costs for CHD and stroke	Underlying cause of hospitalisation (2018)	Average cost of hospitalisations per individual	National Hospital Information System (SIH/SUS) [26]
	Primary health, outpatient and informal care and medication costs for CHD and stroke		Costs were extrapolated to Brazilian settings	Leal et al. [27]

The model is designed first to run the no intervention scenario, simulating the life courses of the synthetic individuals and recording sodium consumption, systolic blood pressure, first cardiovascular episode, and death (from CVD or any other cause). Then, it simulates the life courses of the same synthetic population under the intervention scenario (in this case, food reformulation through the national voluntary sodium targets) and records its outcomes.

The close-to-reality population built in the model mirrors the Brazilian national population structure by age and sex [18]. It includes data from the National Health Survey (*Pesquisa Nacional de Saúde—*PNS 2013) [22] and the National Household Budget Survey (*Pesquisa de Orçamentos Familiares—*POF 2008–2009) [20, 21] regarding sodium and systolic blood pressure (SBP) exposure.

### Microsimulation model structure

**Fig. 1:**
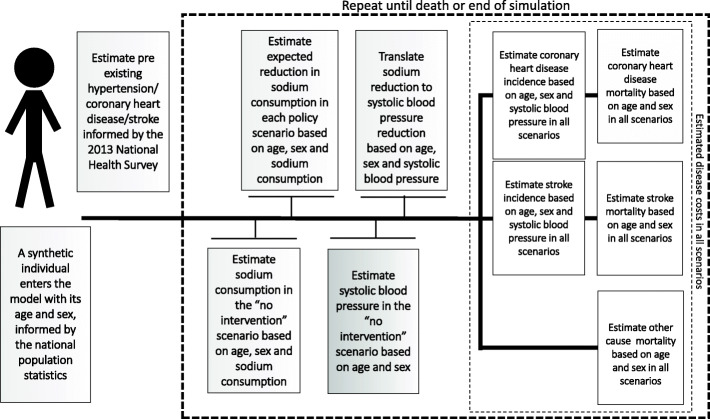
Simplified structure of the Brazil IMPACT Sodium Policy Model

Figure 1 shows a simplified structure of the IMPACT_NCD-BR_ model. The model tracks individual-level sodium consumption, considering the different sources of dietary sodium, its impact on SBP, and the consequent risk of developing CHD, stroke, and death from these or any other cause. IMPACT_NCD-BR_ is calibrated to forecasts of CHD, stroke, and any-other-cause mortality for the whole Brazilian population from 2013 to 2032. The results are presented for adults aged 30 to 79 years within the simulation horizon of 20 years.

For this, the model draws the traits of the synthetic individuals from conditional distributions, projects the sodium intake into the future, and uses the projections to evolve the traits of the synthetic individuals over time. We used PNS 2013 for the SBP projections (assuming SBP remains constant over time for all age and sex groups) and POF 2008–2009 for the sodium intake projections [20–22].

### Summary of evidence regarding the risks of excess sodium consumption

Excess dietary sodium consumption has been linked to an increased risk of CVD [28]. For CVD, the excess risk appears to be mainly mediated through the deleterious effect of excess sodium consumption on blood pressure (BP) [3]. Our methods for evaluating the causality of sodium reduction on BP and BP reduction on CVD have been previously described [3].

### Policy effects

Brazil’s Ministry of Health has set voluntary reduction targets with food industries, from 2011 to 2017, based on upper bound sodium concentration targets for 34 food categories [11, 29]. In addition, publicly available data from the Household Budget Survey (POF 2008–2009) were used to map these 34 food categories through household food acquisition [20] and a 24-h recall dietary questionnaire [21]. These data enabled the model to estimate the potential impact of the modelled policies on every synthetic individual based on their age, sex, and sodium consumption in the no intervention baseline scenario. The model then used the estimated reduction in sodium consumption of the synthetic individuals to calculate the effect upon their SBP using a published meta-regression equation [3].

We assumed that reformulation of food products would adjust sodium content to targets until 2017 and that this would lead to an immediate change in sodium intake in synthetic individuals according to the level of reformulation. We also assumed that the reformulated products would, after that, sustain their sodium concentration.

Although changes in sodium intake influence SBP within weeks [3, 30], we conservatively assumed a median duration of 5 years from a change in SBP to the health outcomes of CVD cases and CVD deaths.

### Modelling of food composition and sodium intake changes

We have considered changes in food composition from the voluntary sodium targets using data from official national food labelling surveys in 2013 and 2017: the baseline targets and the most recent documented official monitoring, respectively [12]. In addition, changes in sodium intake were modelled using microdata from the 2002–2003 and 2008–2009 Brazilian Household Budget Survey. We assumed that the average food consumption and use of table salt in the population remained constant from 2011 to 2017 and that sodium content was reduced for the targeted food categories only by industries that have voluntarily committed to the national sodium targets (which correspond to a 70% market share in the country).

We used the publicly available data from the Household Budget Surveys (POF) of 2002–2003 [31] and 2008–2009 [20] in order to estimate the changes in sources of dietary sodium (non-industrial and industrial sources) during the period between both surveys. We projected the continuity of the replacement of discretionary salt (table salt) by processed and ultra-processed foods (representing the other sodium sources), assuming that the replacement would continue at the same rate in the future.

### Model outputs

The model generates the total numbers of relevant events and reported case-years (CHD and stroke) and deaths prevented or postponed (CHD or stroke or other) for each scenario. We present the results for Brazilian adults aged 30 to 79 years from 2011 to 2032 (simulation horizon of 20 years), rounded to 2 significant digits. For simplicity, we used the term “cases prevented or postponed” to express case-years prevented or postponed.

### Medical costs analyses

The CHD and stroke hospitalisation costs to the Brazilian National Health System (SUS—*Sistema Único de Saúde)* were obtained from the publicly available tables from the Hospital Information System—SIH-SUS [26]. There were no readily available Brazil-specific data for the informal care costs; therefore, we used estimates for these costs from a study on European Union (EU) countries [27]. Cost savings to the health system and the population were estimated considering the CHD and stroke cases prevented or postponed and the average costs for a person-year living with these diseases. Costs were collected in Brazilian Reals (R$) and subsequently converted to US dollars (US$), at an exchange rate of US$1 = R$3.876, current as of December 31, 2018, as reported by the Central Bank of Brazil, and all costs and were discounted at a 3% annual rate.

### Sensitivity analyses and uncertainty analyses

We performed probabilistic sensitivity analyses using a second-order Monte Carlo approach for estimating the uncertainty of different model parameters and population heterogeneity to be propagated to the outputs [32]. Uncertainty was based on sources of the sampling errors of baseline sodium intake, baseline SBP, and the relative risk of CHD and stroke based on SBP, the uncertainties around the lowest sodium and SBP exposures below which no excess risk is observed, around the effect of sodium on SBP, around the lag between SBP changes and CVD risk changes, around the true incidence of CHD and stroke, and the uncertainty of mortality forecasts. The output distributions produced through the Monte Carlo approach were summarised by reporting the medians and 95% uncertainty intervals (UIs) using the 2.5% and 97.5% percentiles of the output distribution.

## Results

### Health-related outcomes

**Fig. 2:**
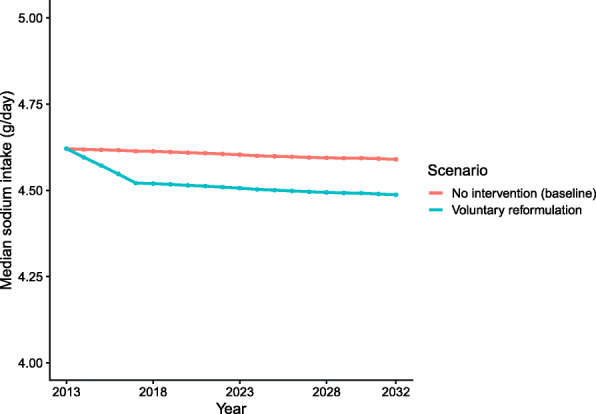
Median daily sodium consumption by year for the two scenarios. Note that the *y* axis does not start from 0

The modelled results suggest that Brazil’s recent voluntary sodium reduction policies have led to a 0.1 g/day of sodium decline in sodium consumption (0.25 g/day salt) from 2011 to 2017 (Fig. 2). If that 0.1g/day sodium reduction continues, it could reduce median SBP from 127.7 mmHg (95% UI: 127.6 to 127.8) in the baseline scenario to 127.5 mmHg (95% UI: 127.4 to 127.6) in the reformulation scenario by 2032. Furthermore, it could prevent or postpone approximately 2500 CVD deaths (95% UI: −900 to 11,000) and some 180,000 CVD cases (95% UI: 45,000 to 430,000), as well as 12,000 deaths from other hypertension-related causes (95% UI: 3600 to 22,000), from 2013 to 2032 (Table 2).

**Table 2:** Health-related model estimates over a 20-year simulation period, from 2013 to 2032, for Brazilian adults aged over 30 years. Brackets contain 95% uncertainty intervals

Outcome	Men	Women	Persons	As percentage of total cases/deaths
CHD cases prevented or postponed	67,000 (17,000 to 160,000)	31,000 (6000 to 78,000)	98,000 (23,000 to 240,000)	0.072% (0.030 to 0.15%)
Stroke cases prevented or postponed	45,000 (11,000 to 100,000)	39,000 (10,0 00 to 89,000)	84,000 (22,000 to 190,000)	0.14% (0.068 to 0.23%)
CHD deaths prevented or postponed	700 (−200 to 900)	500 (−200 to 2000)	1200 (−500 to 5000)	0.064% (0.000 to 0.15%)
Stroke deaths prevented or postponed	700 (−200 to 3000)	700 (−200 to 2800)	1400 (−500 to 6000)	0.12% (0.039 to 0.23%)
Non-CVD deaths prevented or postponed	6900 (2300 to 13,000)	4600 (1400 to 9200)	12,000 (3700 to 22,000)	0.069% (0.041 to 0.11%)
All deaths prevented or postponed	8300 (1800 to 19,000)	5800 (900 to 14,000)	14,000 (2700 to 33,000)	0.073% (0.045 to 0.11%)

The health benefits of sodium reduction, especially in reducing CHD, were larger in men than women, reflecting men’s higher sodium consumption and higher CHD and stroke burden. In total, during the 20-year time period from 2013 to 2032, the voluntary sodium targets could prevent or postpone approximately 110,000 CVD cases (95% UI: 28,000 to 260,000) in men and some 70,000 cases in women (95% UI: 16,000 to 170,000), and approximately 1400 fewer CVD deaths in men (95% UI: −500 to 4100) and 1200 fewer deaths in women (−500 to 4800).

Almost two-thirds of the CVD cases and CVD deaths that would be prevented or postponed by this policy between 2013 and 2032 would be in people aged 50 to 69 years (Appendix).

### Costs of CHD and stroke

From the public healthcare perspective, the voluntary targets for sodium could result in a net saving of approximately US$ 290 million (95% UI: US$ 71 to 690 million) in cumulative hospitalisation, primary health, outpatient, pharmaceutical, and informal care costs, over the 20 years. Most of the savings would be related to CHD (~75%) rather than stroke.

The US$ 290 million estimated savings through the continuity of the voluntary sodium targets would come from (Table 3):

- Approximately US$ 220 million savings (95% UI: US$ 54 to 520 million) related to reduced CHD and stroke treatment costs to the Brazilian National Health System.
- Approximately US$ 70 million (95% UI: US$ 17 to170 million) savings in informal care costs.

**Table 3:** Impact inventory and cost analysis of CVD-related model outputs for individuals aged 30 to 79 years, assessed cumulatively over the 20-year simulation period from 2013 to 2032 (US$ millions, except when percentages). Brackets contain 95% uncertainty intervals

Outcome	Men	Women	Persons	As percentage of total costs
Change in total health-related costs	190 (48 to 440)	100 (23 to 250)	290 (71 to 690)	0.094% (0.055 to 0.17%)
Total medical costs to SUS	140 (36 to 330)	78 (17 to 190)	220 (54 to 530)	As above
Total informal care costs	46 (12 to 110)	25 (6 to 61)	71 (17 to 170)	As above
Total CHD-related costs	150 (38 to 350)	70 (14 to 180)	220 (52 to 530)	0.072% (0.030 to 0.15%)
CHD medical costs to SUS	120 (29 to 270)	54 (11 to 130)	170 (40 to 400)	As above
Informal care CHD costs	36 (9 to 84)	17 (3 to 42)	52 (12 to 130)	As above
Total stroke-related costs	38 (10 to 86)	33 (9 to 76)	72 (19 to 160)	0.14% (0.068 to 0.23%)
Stroke medical costs to SUS	28 (7 to 64)	25 (7 to 56)	53 (14 to 120)	As above
Informal care stroke costs	10 (3 to 22)	9 (2 to 20)	19 (5 to 42)	As above

## Discussion

We have identified potentially large future health and economic benefits if Brazil were to continue the voluntary sodium targets first set in 2011. We used a previously validated microsimulation approach to create a close-to-reality synthetic population (IMPACT_NCD-BR_ model) for the Brazilian population. Our analysis suggests that continuing the voluntary targets could substantially decrease the CVD burden while also offering considerable cost savings to the public healthcare system and individuals.

However, despite the estimated impacts of the implemented voluntary reformulation, mean sodium intake in Brazil remains well above national targets and WHO recommendations. Hence, there are substantial health and economic opportunity costs of inaction, and despite contributing to the reduction of CVD burden, the existing voluntary targets on processed and ultra-processed foods could be more stringent. Achieving the WHO recommended targets will require a more effective comprehensive strategy, including reformulation, to promote more significant sodium reduction through the whole spectrum of dietary sodium sources in the Brazilian population.

When compared to existing regional and global benchmarks for sodium reduction, Brazil can expect to materialise further benefits by optimising its sodium reduction strategy [29, 33, 34]. The estimated 0.1-g reduction in daily sodium consumption from 2011 to 2017 achieved by Brazil’s voluntary targets has been modest compared with some other countries. A systematic review of 70 papers concluded that multi-component strategies involving both upstream and downstream interventions generally achieved the most considerable reductions in sodium consumption across an entire population, most notably 1.6g/day in Finland and Japan, 1.2g/day in Turkey, and 0.52g/day recently in the UK [9]. Mandatory reformulation alone could achieve a reduction averaging around 0.58g/day (three separate studies), followed by voluntary reformulation (−0.32g/day), school interventions (−0.28g/day), short-term dietary advice (−0.24g/day), and nutrition labelling (−0.16g/day). Tax and community-based counselling could each typically reduce sodium intake by 0.12g/day, while even smaller population benefits were derived from health education media campaigns (−0.04g/day). Although long-term dietary advice could achieve a −0.8g/day reduction under optimal research trial conditions, smaller reductions might be anticipated in unselected individuals [9]. Furthermore, the burden of disease attributable to excessive dietary sodium remains large, with almost 30,000 avoidable CHD and stroke deaths annually [6].

The voluntary targets reduced the average sodium consumption by 0.1 g/day of sodium (0.25g/day salt) between 2013 and 2017 (a 25 mg/day reduction in sodium every year), a reduction of just 2%. By contrast, the UK achieved an average annual reduction of 80 mg/day among men and 50 mg/day among women [14] between 2003 and 2010. However, in high-income countries such as the UK, some 80% of dietary sodium comes from processed and ultra-processed foods, compared with only about 35% in Brazil, whereas over 55% comes from added table salt [7, 20].

The Brazilian voluntary sodium targets have resulted in a gradual sodium reduction to avoid noticeable changes in the taste of foods and allow industries to develop new technologies to reduce or replace sodium [35, 36]. Gradual reductions are unlikely to result in compensatory behaviours as adding more table salt to foods or while cooking [37, 38], while large reductions in a short period may trigger rejection or compensatory behaviour by consumers [39].

In 2018, the attributable costs of hypertension to the Brazilian National Health System reached approximately US$ 525 million/year [40], and in 2013, it was estimated that some US$ 100 million/year in public hospitalisations could be saved if sodium consumption were reduced to 2 g/day in Brazil [41].

This study is focused solely on measuring industrial sodium. This will become increasingly important given the increasing contribution of ultra-processed foods in diets. Food reformulation and other regulatory strategies will, therefore, become essential for improving the food environment by providing healthier food options [9].

Furthermore, reducing future population sodium intake in the Brazilian context will depend on strengthening industry action on sodium and improving strategies to modify population behaviour concerning discretionary (non-industrial) salt use. Thus, the Brazilian Dietary Guidelines emphasise the importance of unprocessed and minimally processed foods as the foundation of diets and conscious use of culinary ingredients like salt.

### Strengths and limitations of this study

The IMPACT_NCD_ microsimulation framework has been extensively validated and replicated in different countries [13, 15]. This is the first IMPACT family microsimulation model adapted to a Latin American country and represents a big step forward for using models to inform policy in the region. The study’s strengths also include the use of representative population data and suitable effect sizes from meta-analyses.

This study also has potential limitations. First, the model’s effect estimates are based on interventional and prospective observational studies and may retain possible biases and confounding factors. However, the etiological effects of dietary changes were estimated from meta-analyses with confirmatory validity analyses, including from randomised clinical trials. Second, the estimated benefits from the model may be conservative and underestimate the full health and economic health gains from sodium reformulation, as (1) the counterfactual scenario assumed that the participation of processed and ultra-processed foods in sodium intake would not change into the future; (2) the model only evaluated diseases mediated through BP, while decreasing sodium consumption could have beneficial effects upon other health burdens such as gastric cancer [42]; (3) it was assumed a median duration of 5 years from changes in SBP to CVD risk; (4) only the first cardiovascular episode was considered. Therefore, only primary prevention benefits were quantified; and (5) food reformulation by industries might additionally increase potassium intake through substitution of NaCl with KCl [43], which potential beneficial effect was not included in the model. Third, informal costs were based on evidence from European countries and, therefore, might not represent Brazil’s informal care costs. Finally, medical costs from the private sector, which covers 30% of the Brazilian population, were not included in the economic estimates, which will therefore be conservative.

## Conclusions

Our findings suggest that the voluntary sodium reduction targets for processed and ultra-processed foods in Brazil could generate health benefits to the population and cost savings to the National Health System and informal health treatment cost. Nevertheless, to achieve more substantial sodium reduction and its consequent health and economic impacts, lower sodium targets must be implemented across all food industries. Other dietary sources of sodium must also be tackled, primarily through food-based dietary guidelines and comprehensive approaches to healthy diets. The potential health and economic benefits could be substantial.

## Supplementary Information

**Additional file 1: **High-level description of the IMPACT_NCD-BR_ model. **Figure 1.** A logical framework for the IMPACT _NCD-BR_ model. **Figure 2.** Coronary heart disease mortality by sex. Observed versus modelled number of deaths. **Figure 3.** Stroke mortality by sex. Observed versus modelled number of deaths. **Figure 4.** Other (non-coronary heart disease, non-stroke) mortality by sex. Observed versus modelled number of deaths. Figure 5. Population size by sex. Observed versus modelled number of deaths. **Table S1.** The IMPACT_NCD-BR_ model data sources. **Table S2.** Key modelling assumptions and limitations. **Table S3.** Food categories in the Brazilian voluntary agreements and their mean sodium content at the baseline of negotiations and in 2017 (mg/100g). **Table S4.** Sodium intake by age and sex groups and by dietary sodium sources at baseline (POF 2008-2009 survey). **Table S5.** Estimated sodium intake by age and sex groups and by dietary sodium sources considering food reformulation caused by the Brazilian voluntary target scenarios (2017). **Table S6.** Health-related model estimates over the 20-year simulation period from 2013 to 2032 for Brazilian adults age 30 to 79 years by sex [3, 4, 7, 11–15, 18, 19, 24, 26, 27, 29, 32, 44–81].

## Data Availability

All relevant data are within the paper, supporting information files, and the GitHub repository (https://github.com/ChristK/IMPACTncd_Br/tree/voluntary_reformulation). The source code of the model is available at https://github.com/ChristK/IMPACTncd_Br/tree/voluntary_reformulation. All data inputs are available as .csv files in this repository, except the Population, Household Budget and Health Survey for Brazil microdata, which are publicly accessible through the Brazilian Institute of Geography and Statistics (https://www.ibge.gov.br/), and the hospitalisation and mortality data, which is also publicly available through the Brazilian National Health System’s Department of Informatics (http://tabnet.datasus.gov.br/).

## Abbreviations

BP: Blood pressure
CHD: Coronary heart disease
CVD: Cardiovascular disease
DALYs: Disability-adjusted life-years
NCD: Non-communicable disease
PNS: National Health Survey
POF: Household Budget Survey
SBP: Systolic blood pressure
SIH-SUS: Hospital Information System
SIM: Mortality Information System
SUS: Brazilian National Health System
UI: Uncertainty interval
WHO: World Health Organization
